# Nightmares and bad dreams preceding suicidal behaviors

**DOI:** 10.1192/j.eurpsy.2023.610

**Published:** 2023-07-19

**Authors:** P. A. Geoffroy, R. Borand, M. Ambar Akkaoui, J. Maruani, M. Lejoyeux

**Affiliations:** Université Paris Cité, Paris, France

## Abstract

**Introduction:**

Sleep and circadian markers are of increasingly growing interest to help better predicting suicide. Indeed, specific sleep disorders and circadian rhythm disorders have been associated to suicidal behaviors, and the most replicated findings are insomnia and nightmares. Nevertheless, these interesting preliminary studies did not examine the chronology and trajectories of these dream contents’ alterations before a suicidal crisis.

**Objectives:**

In this context, we decided to perform a naturalistic study to better understand the characteristic of this population during suicidal crisis, and to evaluate their past dream contents. We aimed to distinguish in this phenomenology three different experiences: bad dreams, nightmares (i.e. awakening bad dreams), and suicidal scenarii during dreams. We hypothesized that these dream experiences may have different chronologies of emergence, and that individuals with dream alterations, compared to individuals without dream alterations, present with different clinical characteristics.

**Methods:**

This naturalistic study included individuals hospitalized between January 2021 and May 2021 in a psychiatric post emergency unit for suicidal crisis (thoughts and attempts).

**Results:**

The study observed that 80% (n=32/40) of patients had altered dreams (AD) before the suicidal crisis, including 27 (67.5%) with bad dreams, 21 (52.5%) with nightmares -awakening bad dreams- and 9 (22.5%) with suicidal scenarii during dreams. No differences were observed between the AD group versus no altered dream (ND) regarding sociodemographic characteristics. We observed a progression of dream content alterations: bad dreams appear 111 days (4 months) before the suicidal crisis, then nightmares appear 87.3 days before (3 months), and suicidal scenarii were reported 45.2 days before (1.5 month). For the AD and ND population in suicidal crisis, 80% met at least one subtype of dream alterations, 40% had bad dreams and nightmares, and 17.5% had the 3 subtypes. The AD group, compared to ND, had significantly more family history of insomnia (p=0.046). Almost all patients (97.5%) had depressive symptoms (MADRS≥7; 82.5% had moderate to severe symptoms, MADRS≥20), 60% had insomnia (ISI>14), 92.5% had altered sleep quality (PSQI>5), and 57.5% reported sleepiness (ESS>10).

**Conclusions:**

Dream alterations and their progression could be readily assessed and may help to better identify prodromal signs of suicidal behaviors.

**Disclosure of Interest:**

None Declared

